# Effects of Leaching Behavior of Calcium Ions on Compression and Durability of Cement-Based Materials with Mineral Admixtures

**DOI:** 10.3390/ma6051851

**Published:** 2013-05-07

**Authors:** An Cheng, Sao-Jeng Chao, Wei-Ting Lin

**Affiliations:** 1 Department of Civil Engineering, National Ilan University, 1 Shen-Lung Road, Ilan 260, Taiwan; E-Mails: ancheng@niu.edu.tw (A.C.); chao@niu.edu.tw (S.-J.C.); 2 Institute of Nuclear Energy Research, Atomic Energy Council, Executive Yuan, Taoyuan 325, Taiwan

**Keywords:** leaching, calcium ion, durability, cement-based materials, mineral admixture

## Abstract

Leaching of calcium ions increases the porosity of cement-based materials, consequently resulting in a negative effect on durability since it provides an entry for aggressive harmful ions, causing reinforcing steel corrosion. This study investigates the effects of leaching behavior of calcium ions on the compression and durability of cement-based materials. Since the parameters influencing the leaching behavior of cement-based materials are unclear and diverse, this paper focuses on the influence of added mineral admixtures (fly ash, slag and silica fume) on the leaching behavior of calcium ions regarding compression and durability of cemented-based materials. Ammonium nitrate solution was used to accelerate the leaching process in this study. Scanning electron microscopy, X-ray diffraction analysis, and thermogravimetric analysis were employed to analyze and compare the cement-based material compositions prior to and after calcium ion leaching. The experimental results show that the mineral admixtures reduce calcium hydroxide quantity and refine pore structure through pozzolanic reaction, thus enhancing the compressive strength and durability of cement-based materials.

## 1. Introduction

When a cement-based material experiences humidity for a long time period, water may penetrate into the material, causing leaching of calcium hydroxide. Taiwan is located in a subtropical and rainy zone, and thus, cement-based material structures are always exposed to high temperature, humidity, and a salty environment. This circumstance guarantees that the ground water and/or surface rainwater permeates into cement-based materials with time. Penetrating water in cement-based materials along the path of capillary pores renders a ubiquitous ion concentration unbalanced. High concentration ions sequentially move forward to low concentration ions, inducing leaching of hydration products [1]. In addition, underground oil storage composed of cement-based materials also requires care against leaching of calcium ions because concentration ions leach out from cement-based materials to soil or rock, which is in contact with ground water. Leaching of calcium hydroxide was found to be a common case in the underground environment as well. For example, Yokozeki *et al.* (2004) carried out leaching experiments to construct a model to evaluate the effects of various factors on the degradation rate, which is considered to be important for evaluating the long-term performance of cement-based materials [2].

During the leaching process, calcium hydroxide was found to be the first hydration product leached from the cement-based material due to its solubility. Calcium hydroxide is slightly soluble, and can be leached out because of enlarging capillary pores [3]. Leaching of calcium ions increases the porosity of cement-based material, thus resulting in degradation including damage to the pore structure. Increasing porosity results in a weakened matrix and lower compressive strength of the cement-based material. Leaching of calcium ions also has a detrimental effect on durability, since the occurrence provides an entry for aggressive harmful ions into the cement-based material, causing reinforcing steel corrosion. While cement-based material is degraded by the leaching of calcium ions, compressive strength drops dramatically. Furthermore, the degradation volume of the cement-based material can be increased with the leaching progression of calcium ions, and has a linear relationship with the loss in compressive strength [4].

Cement-based materials inherit low tensile strength, are brittle, and have other shortcomings. When cement-based materials are exposed to harsh environments, insufficient structural durability is always a common problem. Based on the previous research data, the loss in durability caused structural damage, and repair costs increased yearly in the United States [5]. For example, the repairing costs for reinforcing steel corrosion in U.S. highway concrete bridges caused by salt attacks have been more than $150 billion USD up to the year of 2007 [6]. Studies focusing on lowering the cause of the durability degradation mechanism and exploring influential factors have been extensive. Based on their results, these studies proposed to increase the durability of the structures. However, the indicator of durability has not yet been clearly defined. Thus, establishing objective indicators of durability for proper assessment is necessary to ensure a sustainable design or maintenance [7,8].

The previous studies [9,10] reported that the rate of calcium leaching may be very slow. The leaching depth of concrete submerged in still water for 100 years was about 5 to 10 mm [10]. To speed up and investigate the state of leaching out of calcium ions over time, accelerated leaching test methods are usually adopted, namely, electrochemical and chemical acceleration methods [4,9]. Hiroshi Saito [1] performed leaching tests on different mortars by employing an electrochemical method, which accelerates the dissolution of cement hydrate from mortar in contact with water to apply a potential gradient across the specimen. Jain [11] has dealt with calcium leaching from cement pastes incorporating glass powder, silica fume or fly ash by deionized water medium method. Planel [12] has continuous monitored the calcium loss in pure deionized water and performed the calcium-depleted part of the specimens by microstructure observation. The chemically accelerated method was chosen with the use of a concentrated ammonium nitrate solution. The similarity of water and leaching has been established chemically, mineralogically and mechanically by Carde [13]. Agostini [3] applied a 6 M concentration of ammonium nitrate solution to accelerate the calcium ions leaching out, which increased the calcium ions leaching out rate 300 times compared with natural calcium leaching [14]. Fundamentally, an ammonium nitrate solution to accelerate the leaching process of the cement-based materials could be seemed as an easily, quickly and quantitatively chemical acceleration method.

This study utilized an ammonium nitrate solution to accelerate the leaching process of the cement-based materials. Leaching durations were set to 56 days, 91 days, and 140 days. Scanning electron microscopy, X-ray diffraction analysis, and thermogravimetric analysis were employed to analyze and compare the cement-based material compositions prior to and after calcium ions leaching out. In addition, leaching depth, initial surface absorption test (ISAT), mercury intrusion porosimetry (MIP), and ultrasonic pulse velocity were measured to analyze the effects of calcium ions leaching on the pore structures of the cement-based materials. The effect of calcium ions leaching on the compressive strength and durability of cement-based materials with mineral admixtures can thus be investigated via chemical analysis and pore structure analysis.

This study investigated the effects of leaching behavior of calcium ions on compressive strength and durability of the cement-based materials. Since the parameters influencing the leaching behavior of cement-based materials are unclear and diverse, this study focused on the influence of adding mineral admixtures (fly ash, slag, and silica fume) on the leaching behavior of calcium ions regarding compression and durability of cemented-based materials. The effects of calcium ion leaching on the compressive strength and durability of cement-based materials with mineral admixtures were investigated via chemical analysis and pore structure analysis.

## 2. Experimental Program

### 2.1. Material Properties

#### 2.1.1. Fine Aggregate

The fine aggregate used in this study are the sand deposits of Langyang River, Taiwan. The physical properties of the Langyang River sand are listed in Table 1.

**Table 1:** The physical properties of the Langyang River sand.

Test	Designation	Value
Specific gravity (SSD)	CNS 487	2.62
Absorption (%)	CNS 487	1.58
Fineness Modulus (FM)	CNS 486	2.82

#### 2.1.2. Cement and Mineral Admixtures

Type I Portland cement produced by the Taiwan Cement Corporation was used for all the specimen mixtures. The chemical compositions of cement and mineral admixtures are summarized in Table 2.

**Table 2:** The physical and chemical properties of Portland cement type I and mineral admixtures.

Chemical composition	Slag	Fly ash	Silica fume	Portland cement
SiO_2_ (equiv %)	33.5	54.0	91.5	21.2
Al_2_O_3_ (equiv %)	9.0	24.0	0.2	5.4
Fe_2_O_3_ (equiv %)	3.6	8.0	0.7	3.2
CaO (equiv %)	43.8	2.0	0.4	63.8
MgO (equiv %)	2.7	1.3	1.5	2.0
K_2_O+Na_2_O (equiv %)	0.6	0.9	1.9	0.8
Other (equiv %)	6.8	9.8	3.8	3.6
**Physical properties**	**Slag**	**Fly ash**	**Silica fume**	**Portland cement**
Specific gravity	2.89	2.46	2.20	3.15
Surface area (m^2^/kg)	415	420	22500	364

### 2.2. Mix Design of the Cement-Based Material

The specimens prepared for this study are based on the requirements of ASTM C109. The water/binder (w/b) ratio is fixed at 0.55 for all specimens in this study, while the cement/sand ratio is set at 1:3.

Type B specimen represents cement-based material without replacing with any mineral admixture in this paper. Type G20, G40, and G60 represent the specimens replacing Portland cements with slag by 20%, 40%, or 60% by weight, respectively. Type F10, F20, and F30 represent the specimens replacing Portland cements with fly ash by 10%, 20%, or 30% by weight, respectively. Type S5, S10, and S15 represent the specimens replacing Portland cements with silica fume by 5%, 10%, or 15% by weight, respectively. The mix proportions for the abovementioned specimens are displayed in Table 3, Table 4, Table 5 and Table 6.

**Table 3:** Mix design of cement without mineral admixture—Type B specimen.

**Specimen**		B
**Water/binder (w/b)**		0.55
**Component weight**	**Water (kg/m^3^)**	308
	**Cement (kg/m^3^)**	560
	**Fine aggregate (kg/m^3^)**	1680

**Table 4:** Mix design of cement with slag—Type G specimen.

Specimen	Water/binder (w/b)	Component weight			
		Water (kg/m^3^)	Cement (kg/m^3^)	Slag (kg/m^3^)	Fine aggregate (kg/m^3^)
G20	0.55	286	416	104	1560
G40	0.55	286	312	208	1560
G60	0.55	286	208	312	1560

**Table 5:** Mix design of cement with fly ash—Type F specimen.

Specimen	Water/binder (w/b)	Component weight			
		Water (kg/m^3^)	Cement (kg/m^3^)	Fly ash (kg/m^3^)	Fine aggregate (kg/m^3^)
F10	0.55	286	468	52	1560
F20	0.55	286	416	104	1560
F30	0.55	286	364	156	1560

**Table 6:** Mix design of cement with silica fume—Type S specimen.

Specimen	Water/binder (w/b)	Component weight			
		Water (kg/m^3^)	Cement (kg/m^3^)	Silica fume (kg/m^3^)	Fine aggregate (kg/m^3^)
S5	0.55	286	494	26	1560
S10	0.55	286	468	52	1560
S15	0.55	286	442	78	1560

Additionally, the meaning of the specimen numbers X_1_, X_2_, X_3_ is used as follows: First number X_1_: mix type; Second number X_2_: leaching (L); and Third number X_3_: leaching duration (0, 56, 91, or 140 days).

### 2.3. Test Preparation

In this study, researchers cast a total of 400 specimens comprising 10 different mixes. Each testing values reported are the average of five specimens. The 50 mm × 50 mm × 50 mm cubical specimens of each mix were prepared for the compressive strength test, accelerated leaching test, initial surface absorption test and ultrasonic pulse velocity measurement. Specimens with a thickness of 10–15 mm were sliced from cubical specimens and used in mercury porosity test. The 10 mm × 10 mm × 5 mm specimens for scanning electron microscopy (SEM) and X-Ray Diffraction Analysis (XRD) were taken from the surface layer of the slices cut from the cubical specimens. In addition, it prepared 3 g specimens of mortar powders for thermogravimetryanalysis (TGA) testing.

**Figure 1:**
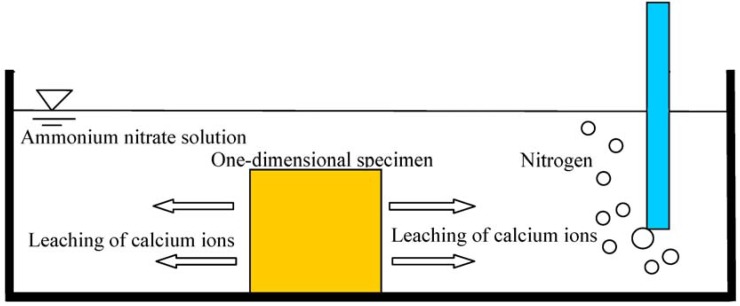
Scheme describing the accelerated leaching protocol using ammonium nitrate solution.

The test specimen for accelerated leaching test is designed as a one-dimensional approach to study the leaching behavior. To control the specimen to a one-dimensional path of leaching out, the other sides of the specimen require sealing with anti-acid adhesive tape. Thus, the effects from the leaching depth to the compressive strength, porosity, wave velocity, and chemical compounds can be explored. The scheme describing the accelerated leaching protocol using ammonium nitrate solution is illustrated in Figure 1.

### 2.4. Test Methods and Procedures

#### 2.4.1. Compressive Strength

This study conducted compressive strength tests in accordance with ASTM C109 at 56 days. Test specimens were prepared by placing the cement-based material into 5 cm × 5 cm × 5 cm molds. After 24 h curing, the specimens were demolded and placed in a water tank at room temperature until testing. The specimens were placed on a universal testing machine with a rate of 1.4–3.4 kgf/cm^2^ of pressure per second to execute the uniaxial compression test.

#### 2.4.2. Accelerated Leaching Test

This experiment required utilizing the chemical solution immersion approach to accelerate the leaching of calcium ions. The aggressive solution was prepared as a concentration of 6 M ammonium nitrate solution (480 g/L), with leaching times at 56, 91, and 140 days. Such a solution leads to acceleration of the leaching process by hundreds of times compared to deionized water. Nitrogen bubbling was used to avoid carbonation. The specimens were moved into deionized water soaking for 10 days before testing for the compressive strength and durability.

#### 2.4.3. Leaching Depth Measurement

The specimens of the cement-based materials after leaching were sprayed on the split surface with the indicating solution with a 0.1 N silver nitrate in accordance with reference to NT BUILD 492. The leaching depth was determined by observing the color changes.

#### 2.4.4. Thermogravimetry Analysis

This test records weight loss during the temperature change process of the specimen based on the experimental purpose by adjusting the warming background environment and the heating rate. Thus, by carefully observing the weight loss, the specific content of the material composition can be determined. According to TGA curves, and semi-isothermal techniques, cement-based material with temperature decomposition can determine each component [15]. A number of studies have shown that at temperature of ~105 °C, capillary water and gel water evaporates, and between 105 and 440 °C, aluminate and sulfoaluminate products, C–S–H gel, and calcium silicate hydrate hydrolyzes, and at 440–580 °C, the material mainly reaches the calcium hydroxide dehydration decomposition stage, and between 580 and 1007 °C is the decomposition temperature of calcium carbonate.

#### 2.4.5. X-ray Diffraction Analysis (XRD)

This study focused on the analysis of compound change resulting from the leaching process of cement-based materials. Since the hydrations of the composite materials are multiphase, their compositions are all compounds, that is, almost no single element structure exists. X-ray diffraction analysis was thus employed to analyze the cement-based materials.

The chemical compounds of the cement-based material were determined with XRD, which was performed on the specimens in the form of ground powder at room temperature and air-dried conditions. The patterns were taken using a Cu-Kα radiation between 20° and 80°, with a scanning speed of 0.5 s/1°. Comparing the analyzed compounds from the XRD intensity diagram with the matching compounds peak value from the computer database can determine the compositions of the compound ingredients.

#### 2.4.6. Scanning Electron Microscopy (SEM)

The Hitachi model S-510 SEM was used for microstructural observation in this experiment. Scanning electron microscopy utilizes an electron gun firing system exciting electron beams to hit cement-based materials. The specimen surface of the cement-based material can animate a reflecting signal, which is sent through a signal amplifier and subsequently to a cathode-ray tube, before being displayed on a screen. The screen can be used to observe the cement-based material by the surface microcrystalline phase.

#### 2.4.7. Initial Surface Absorption Test (ISAT)

The initial surface absorption test was performed according to BS 1881. The tests determined the rate of surface zone water absorption of the mortar samples during a specific period. The specimen of cement-based material contains capillary porosity; hence, water penetrates into the specimen through the surface. The amount of specimen porosity affects the absorption rate of the pore water surface. Therefore, the resulting amount of initial surface absorption test can effectuate determining the amount of pore volume of the cement-based material.

#### 2.4.8. Mercury Porosity Test

The distribution of pore size and pore volume were measured by employing a Micromeritics Autopore 9500 porosimeter. The mercury porosity test was performed according to ASTM D4404. The device characterizes the porosity of the mortar specimen by applying various levels of pressure to a sample that is immersed in mercury. The size of the pore structure is inversely proportional to the pressure required to intrude the mercury. This is called mercury porosimetry, or often also referred to, “mercury intrusion.” The gel pore (<10 nm), the capillary pore (>10 nm), and the total pore size and volume can be obtained by implementing this test.

#### 2.4.9. Ultrasonic Pulse Velocity

**Figure 2:**
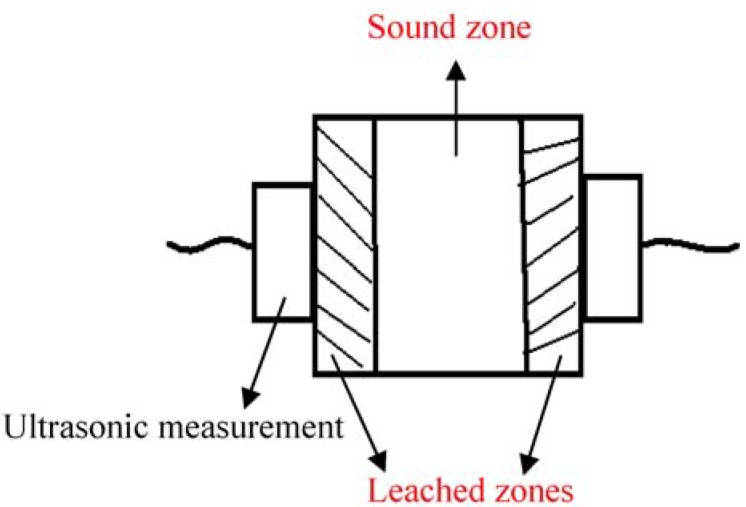
Schematic diagram of an ultrasonic transducer.

This study also measured leaching of cement-based material specimen through the horizontal surface, as shown in Figure 2. The measuring procedure required placing the transducers on both sides of the specimen surface to measure the longitudinal wave velocity through the specimen. This test utilizes ultrasonic pulse velocity measurement travelling between two transducers to explore the influence of the leaching property of different specimens.

## 3. Results and Discussion

### 3.1. Compressive Properties of Cement-Based Material

**Figure 3:**
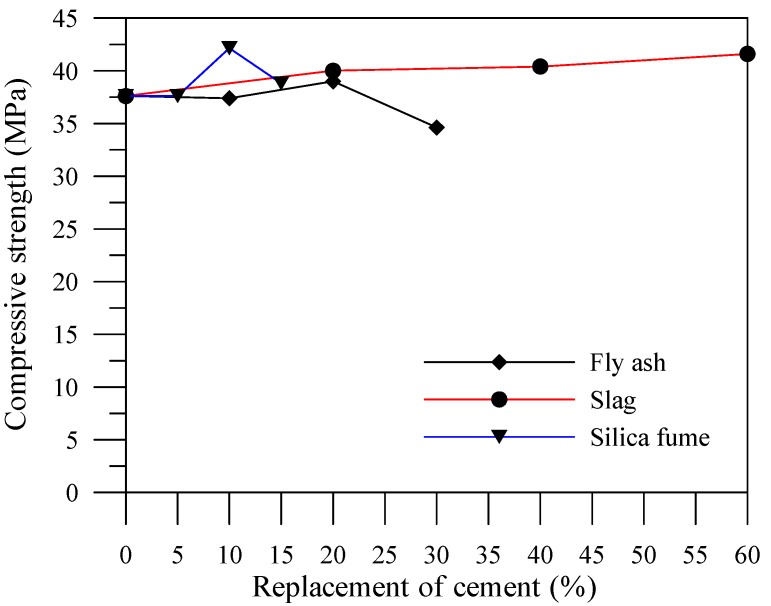
Effects of mineral admixture amount on compressive strengths (56 days).

The relationship of the average compressive strength with the added amount of fly ash for the specimens is shown in Figure 3. The compressive strength of the specimen with 10% fly ash is almost the same as that of the specimen without an admixture. The compressive strength of the specimen with 20% fly ash is higher than that of the specimen without admixtures. Fly ash contains aluminum and silicon; therefore, the hydration process with hydroxide calcium results in a secondary hydration reaction and subsequently produces the C–S–H and C–H–A gel, which increases the compactness of the slag concrete, and enhances its compressive strength. However, the compressive strength of the specimen with 30% fly ash shows a downward trend, and becomes lower than that of the specimen without an admixture. This is because fly ash provides adequate lime required to react with pozzolans in the hydration process. Increased amounts of lime appear to inhibit the hydration process, but are still adequate to provide the required design strength. For the previous study, the compressive strength of fly ash specimens increased with an increase in the number of curing ages [16]. Thus, the cement-based material with the fly ash admixture developed higher compressive strength after 56 days.

The relationship of the average compressive strength with the added slag for the specimens is shown in Figure 3. The compressive strengths of the specimens increased with the addition of slag. Cement hydration reaction produces acidic colloidal film with low permeability on the slag surface in its initial stage. This film prevents the water from contacting and dissolving slag powders. Fortunately, an alkaline environment formed through the dissolving cement hydration process. The alkaline environment stimulates slag activity accelerates slag dissolution. This circumstance results in the slag continuing to consume calcium hydroxide and performing a pozzolanic reaction to produce the C–S–H gel, which can improve the compressive strength of cement-based materials.

The relationship of the average compressive strength with the added amount of silica fume for the specimens is shown in Figure 3. The compressive strength of the specimen with 5% silica fume is almost the same as that of the specimen without an admixture. The compressive strength of the specimen with 10% silica fume is higher than that of the specimen without an admixture. The compressive strength of the specimen with 15% silica fume shows a downward trend, though still higher than that of the specimen without an admixture. Silica fume contains silicon, and therefore, the hydration process with hydroxide calcium results in a pozzolanic reaction before producing the C–S–H gel as well. The silica fume particle size is extremely small and with a high water demand. Hence, adding an excessive amount of silica fume affects the workability and strength conversely, which causes incomplete reaction due to lack of water.

**Figure 4:**
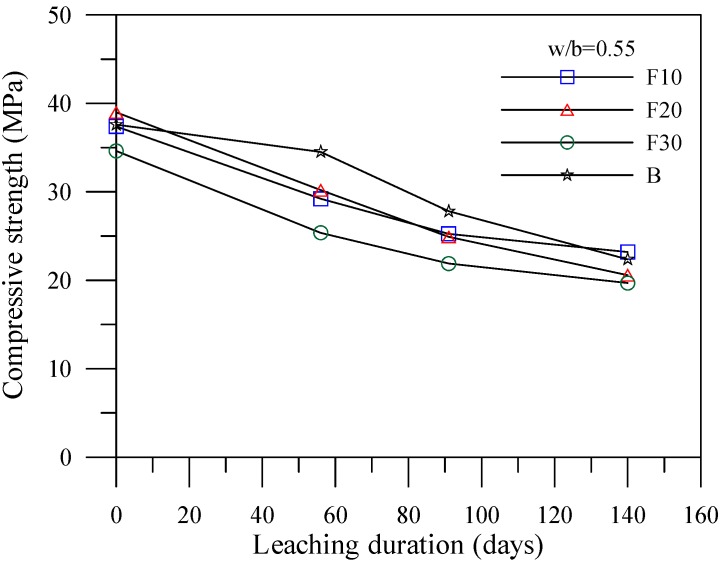
Compressive strength *vs.* leaching duration for the specimens with fly ash.

**Figure 5:**
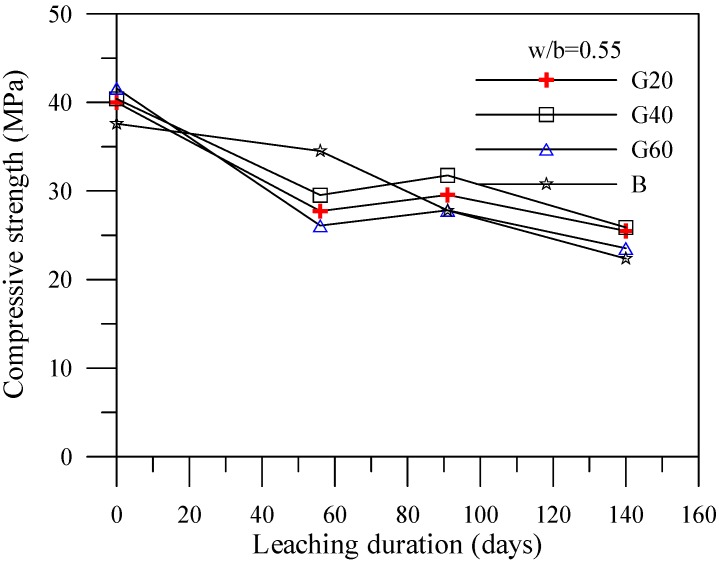
Compressive strength *vs.* leaching duration for the specimens with slag.

**Figure 6:**
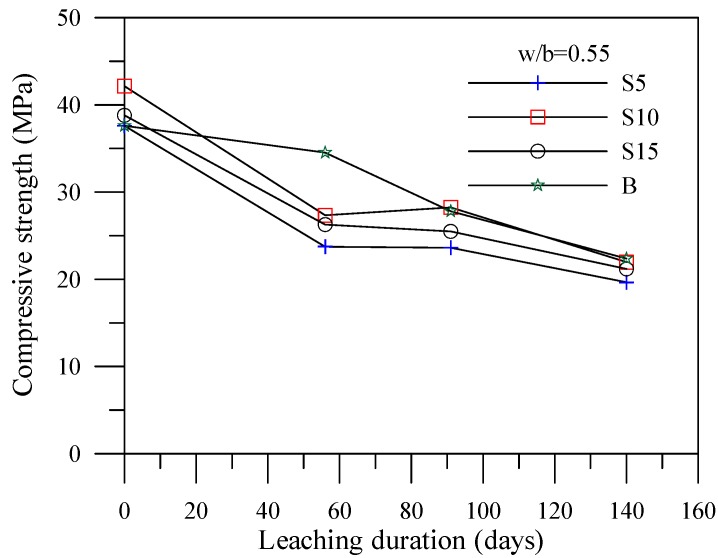
Compressive strength *vs.* leaching duration for the specimens with silica fume.

Figure 4, Figure 5 and Figure 6 illustrate the relationship between compressive strengths and leaching duration for the cement-based materials with various mineral admixtures. The results prior to leaching show that the compressive strength of specimens with mineral admixture tended to increase with the increase of the amount of replacement, which can produce C–S–H gels by consuming calcium hydroxide content. However, the compressive strength significantly decreased with the increase of leaching duration in all mixes. The initial leaching process occurs in the condition of a small amount of C–S–H gel, thus decreasing the compressive strength of the specimens at 56 days. For leaching times at 91 days and 140 days, due to the condition of decreasing particle parking density when calcium ions were leached out, the leaching process of calcium ions decelerates the deterioration of compressive strength of the specimens. This result is consistent with the previous study [17] and the chemical solution immersion leaching testing method is suitable for accelerating the leaching of calcium ions.

**Figure 7:**
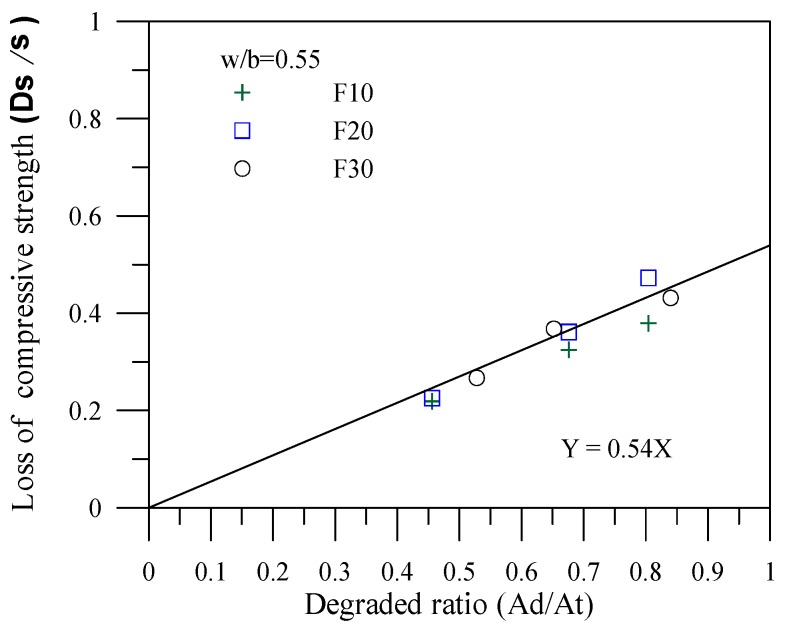
Correlation between the degradation area and the compressive strength loss for the specimens with fly ash.

**Figure 8:**
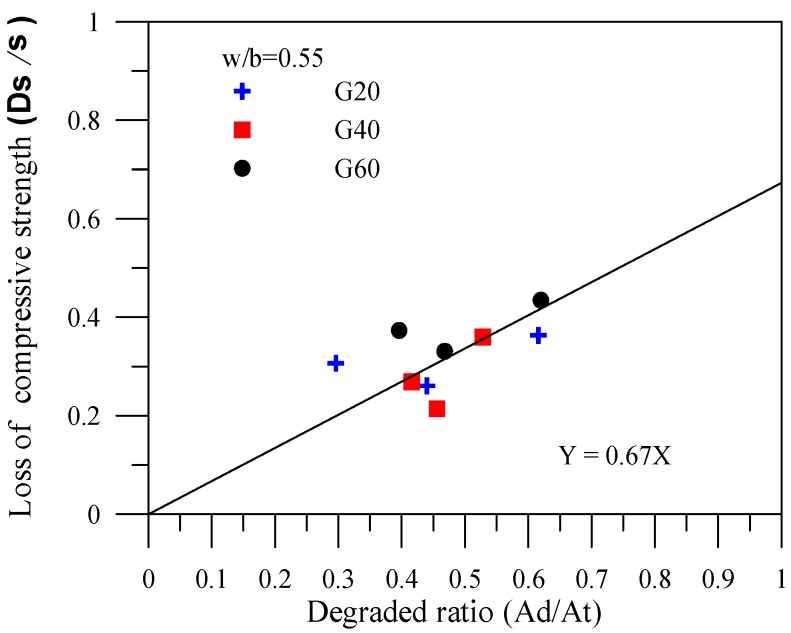
Correlation between the degradation area and the compressive strength loss for the specimens with slag.

**Figure 9:**
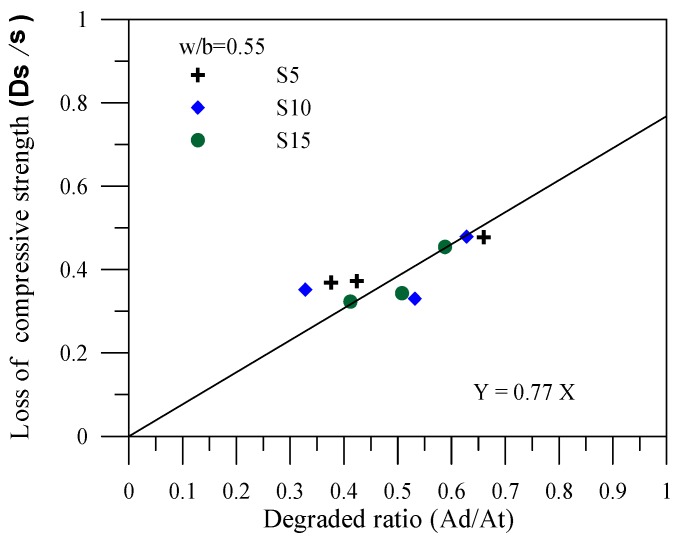
Correlation between the degradation area and the compressive strength loss for the specimens with silica fume.

The correlations between the degradation area ratio and the compressive strength loss ratio for the specimens of various mineral admixtures are shown in Figure 7, Figure 8 and Figure 9. In the figures, σ represents the compressive strength of the specimen with no leaching; $\Delta \sigma \text{(}=\sigma \text{-}\sigma_{\text{c}}\text{)}$ represents the amount of compressive strength loss due to leaching, where $\sigma_{\text{c}}$ is the result of the compressive strength leaching test, and Δσ/σ represents the compressive strength loss ratio of the cement-based material after leaching. In addition, *Ad* in the figures represents the leaching area of the degraded specimens; *At* stands for the total area of the tested specimens. The degradation area ratio can thus be expressed as $A_{d}/A_{t}$. Applying the linear regression technique can effectuate estimation of the compressive strength of the complete leaching deterioration $\sigma_{\text{L}}$. Specimens with fly ash provide a completely degraded strength of $\sigma_{\text{L}}$ = 0.54σ. In other words, a complete deterioration for specimens with fly ash remains at 54% of the original strength. Specimens with slag provide a completely degraded strength of $\sigma_{\text{L}}$ = 0.67σ. Specimens with silica fume provide a completely degraded strength of $\sigma_{\text{L}}$ = 0.77σ, which is the optimal resistance to the degradation of compressive strength.

### 3.2. Leaching Degraded Depth

**Figure 10:**
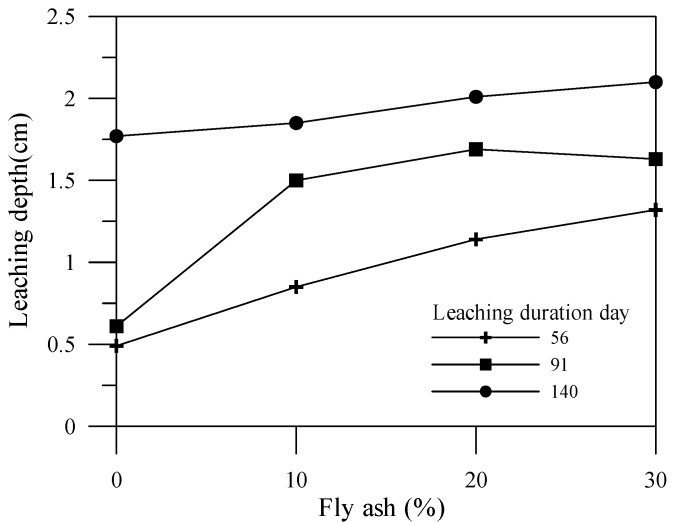
Effects of fly ash admixture amount on leaching depth and duration.

**Figure 11:**
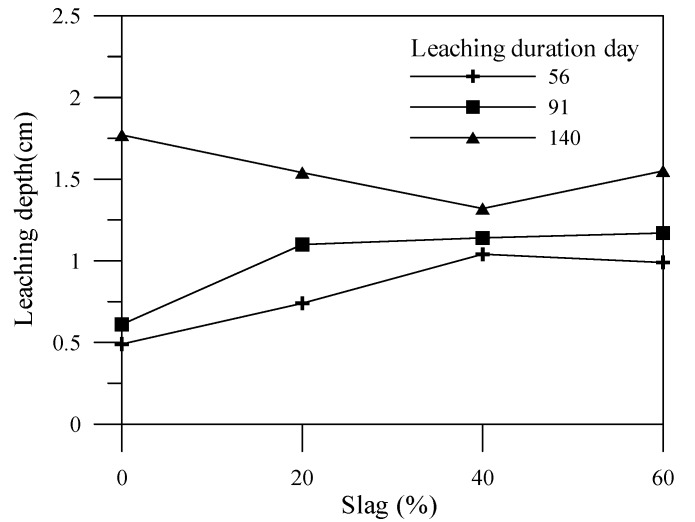
Effects of slag admixture amount on leaching depth and duration.

**Figure 12:**
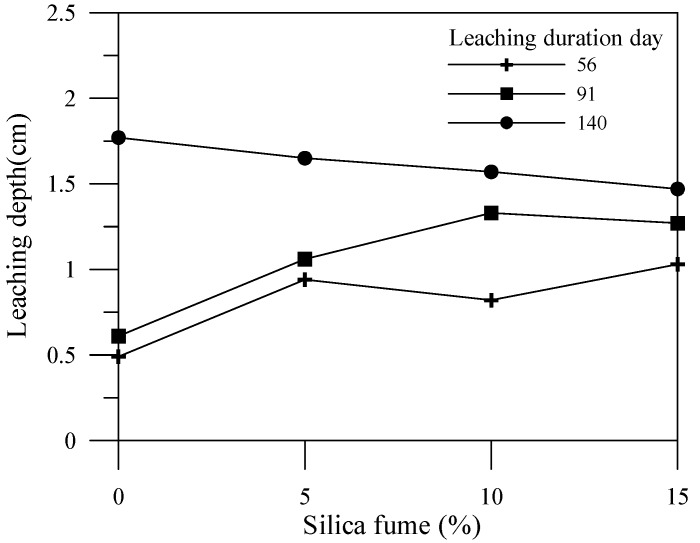
Effects of silica fume admixture amount on leaching depth and duration.

This experiment utilizes the color change of the specimens to determine the leaching depth as shown in Figure 10, Figure 11 and Figure 12 for different leaching durations. Cement is the main source for providing an alkaline environment to the specimen, while the alkaline environment of the specimen would be reduced within the leaching area. Generally, leaching depths of the cement-based material specimens with mineral admixtures at 56 days and 91 days are higher than that of the specimen without mineral admixtures. Nonetheless, leaching depths of the cement-based material specimens with mineral admixtures at 140 days or more are less than that of the specimen without mineral admixtures. This is because the mineral admixture replacing cement results in a post-production pozzolanic reaction, thus producing C–S–H gel, which can improve the specimen density. A previous study [17] indicated that increasing the mineral admixture to reduce the amount of cement would produce C–S–H gels by consuming calcium hydroxide content during the pozzolanic reaction. Therefore, the porosity in the specimen is reduced, and the pore size distribution is also affected. Adding an appropriate amount of mineral admixture facilitates the reduction of the leaching depth of a specimen.

A neutralized cement-based material specimen results in the destruction of the passive film of the reinforced steel to commence corrosion. The leaching depths and times of the specimens can be used to estimate the completely neutralized duration. When calcium ions reach the reinforced steel due to their leaching thickness, this process is defined as the specimen being neutralized. This mechanism can be used to approximate the thickness of reinforcement neutralized duration, which signifies the deterioration of service time for the specimens. In other words, a specimen reaching complete neutralization time is equal to the initiation time for the corrosion of reinforced steel. Assuming that the thickness of a protective layer is 2.5 cm, the complete neutralized duration can be obtained via extrapolation for specimens with various mineral admixtures. The time required to reach complete neutralization was proposed previously, that the specimen leaching depth and the square root of the days have a linear relationship [18].

As stated above; 6 M concentration of ammonium nitrate solution can accelerate the leaching process of the specimens by 300 times [13]. Therefore; the previously predicted accelerated leaching time pass through the protective layer requires multiplication by 300 times for the true neutralized corrosion time of reinforced steel regarding deterioration of the cement-based materials; as shown in Table 7. Concerning the replacement of cement with fly ash at 10%; 20%; and 30%; the deterioration of reinforced steel due to calcium ion leaching were at 234 years; 165 years; and 167 years; respectively. Concerning the replacement of cement with slag at 20%; 40%; and 60%; the deterioration of reinforced steel due to calcium ion leaching were at 362 years; 362 years; and 313 years; respectively. Finally; regarding the replacement of cement with silica fume at 5%; 10%; and 15%; the deterioration of reinforced steel due to calcium ion leaching were at 318 years; 304 years; and 308 years; respectively.

**Table 7:** Predicted leaching time for cement-based materials with mineral admixtures.

Specimen	Prediction equation	Accelerated time (day)	True time (day)	True time (year)
F10	Y = 0.148X (R^2^ = 0.99)	285	85600	234
F20	Y = 0.176X (R^2^ = 0.99)	201	60530	165
F30	Y = 0.175X (R^2^ = 0.99)	204	61224	167
G20	Y = 0.119X (R^2^ = 0.99)	441	132405	362
G40	Y = 0.119X (R^2^ = 0.99)	441	132405	362
G60	Y = 0.128X (R^2^ = 0.99)	381	114440	313
S5	Y = 0.127X (R^2^ = 0.99)	387	116250	318
S10	Y = 0.130X (R^2^ = 0.99)	369	110946	304
S15	Y = 0.129X (R^2^ = 0.99)	375	112673	308

### 3.3. Scanning Electron Microscopy (SEM)

**Figure 13:**
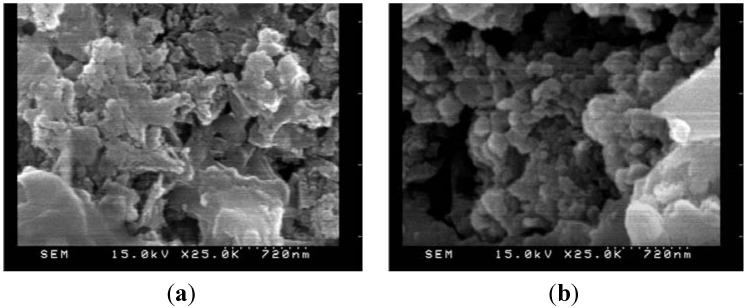
SEM micrograph of the cement-based material with fly ash admixture (×25 k):(**a**) Before leaching; (**b**) After leaching.

The SEM images of cement-based material with fly ash admixture are shown in Figure 13 to demonstrate the crystalline microstructure. Figure 13 displays both the microstructures of the specimens prior to and after the leaching process. In the phase prior to leaching, the hydration products are quite denser inside the specimen. Once the specimen was disintegrated by the chemical reaction of the ammonium nitrate solution, developing soluble calcium nitrate, expansible calcium nitrate, and ammonium, the leaching of hydration products significantly decreased and produced increasing porosity [19]. The specimens of cement-based materials with slag or silica fume share the same phenomenon with fly ash.

### 3.4. X-ray Diffraction Analysis (XRD)

**Figure 14:**
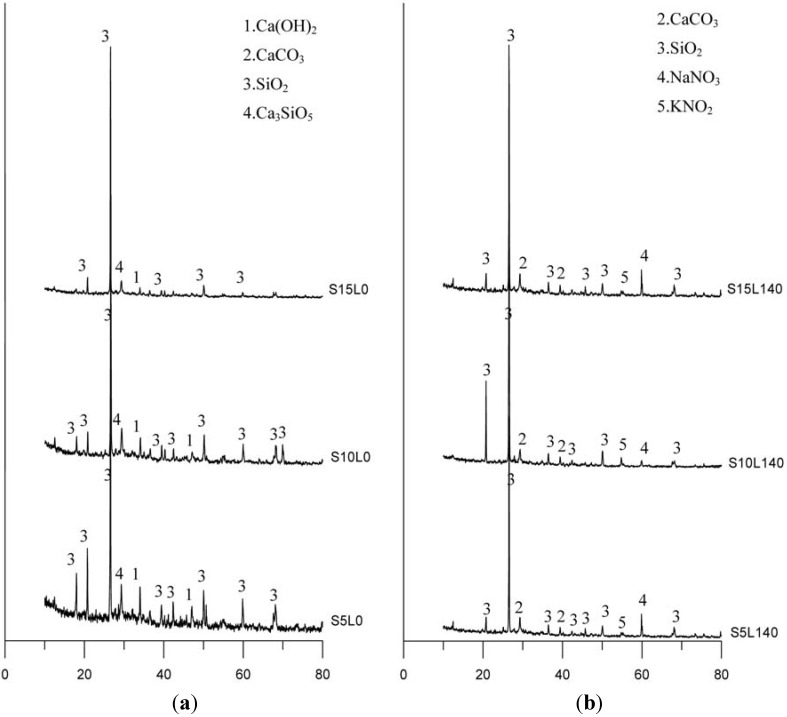
XRD results of specimens with silica fume admixture: (**a**) Prior to leaching; (**b**) After leaching.

The XRD results of the specimens with the silica fume admixture prior to leaching and after leaching are both illustrated in Figure 14. Figure 14a for the specimen before leaching shows that the main peaks corresponded to calcium hydroxide, calcium silicate, and silica chemical composition. Figure 14b shows the specimen after leaching via the XRD accelerated leaching test, and that the specimens in the deterioration portion do not contain a calcium hydroxide component. During the reaction induced by the ammonium nitrate solution, the specimen could have possibly effectuated the gradual leaching out of calcium hydroxide. The hydration products of specimens having chemical reactions with the ammonium nitrate solution formed the degradation portion after leaching, producing chemical products associated with ammonium nitrate, which can be found via XRD, as shown in Figure 14b. Moreover, the specimens of cement-based materials with slag or fly ash demonstrate the same reaction with silica fume.

### 3.5. Thermogravimetry Analysis

**Figure 15:**
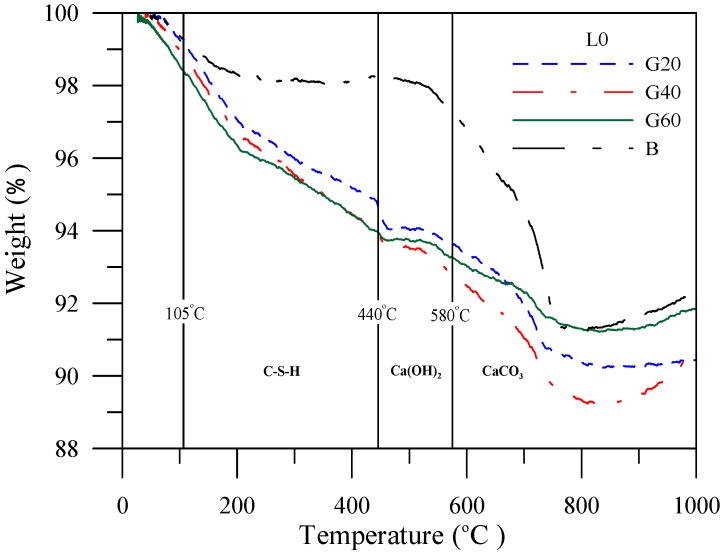
Weight loss of the specimens with slag admixture prior to leaching *vs.* temperature change.

**Figure 16:**
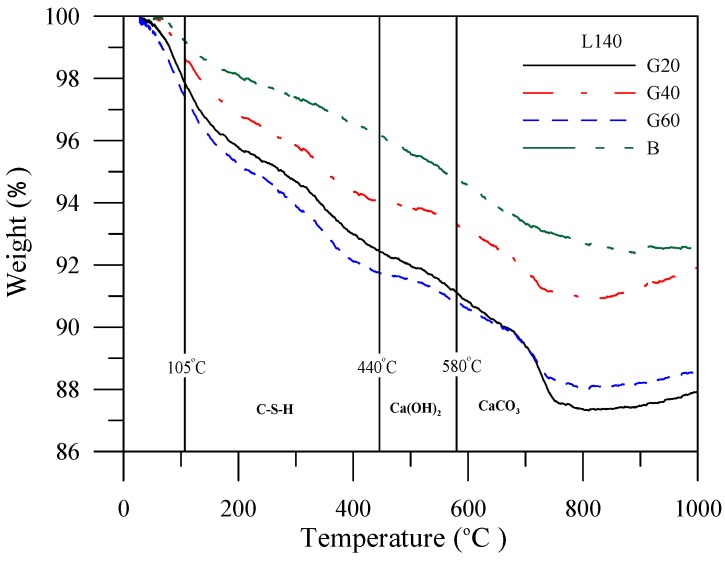
Weight loss of the specimens with slag admixture after leaching *vs.* temperature change.

The TGA curves are represented as the quantitative mass loss of paste products, as shown in four mass loss transitions similar to the previous studies [15]. The weight loss between 25 °C and 105 °C, 105 °C and 440 °C, 440 °C and 580 °C, 580 °C and 995 °C corresponded to the decomposition of water from the capillary and gel pores, the decomposition of water from the hydrated phases, the dehydroxylation of water from calcium hydroxide, the decomposition of calcium carbonate, respectively. The weight loss curves of the specimens with slag admixtures prior to leaching and after leaching are shown in Figure 15 and Figure 16.

The weight loss curves can be obtained under different temperatures before being organized into tables to facilitate analysis and discussion. Table 8 shows that prior to leaching, the specimens with slag admixtures facilitate incrementation of the development of C–S–H gels, and of the decrease in calcium hydroxide content. Table 9 shows that calcium hydroxide content and C–S–H gels in the specimen are reduced, implying that the calcium ions were precipitated from the specimens during the leaching process. Furthermore, the specimens of cement-based materials with fly ash or silica fume share the same phenomenon with slag.

**Table 8:** Weight loss of the specimen with slag admixture under different temperature prior to leaching.

Temperature	Curing period	Weight loss percent (%)			
		BL0	G20L0	G40L0	G60L0
105~440 °C	56 days	1.06	4.37	4.54	4.83
440~580 °C	56 days	1.41	1.24	1.14	0.78
580~1000 °C	56 days	4.77	3.10	2.15	1.32

**Table 9:** Weight loss of the specimen with slag admixture under different temperature after leaching.

Temperature	Curing period	Weight loss percent (%)			
		BL140	G20L140	G40L140	G60L140
105~440 °C	56 days	0.9	2.53	2.44	2.13
440~580 °C	56 days	0.94	1.00	0.74	0.68
580~1000 °C	56 days	2.26	2.51	1.39	1.12

### 3.6. Initial Surface Absorption Test (ISAT)

**Figure 17:**
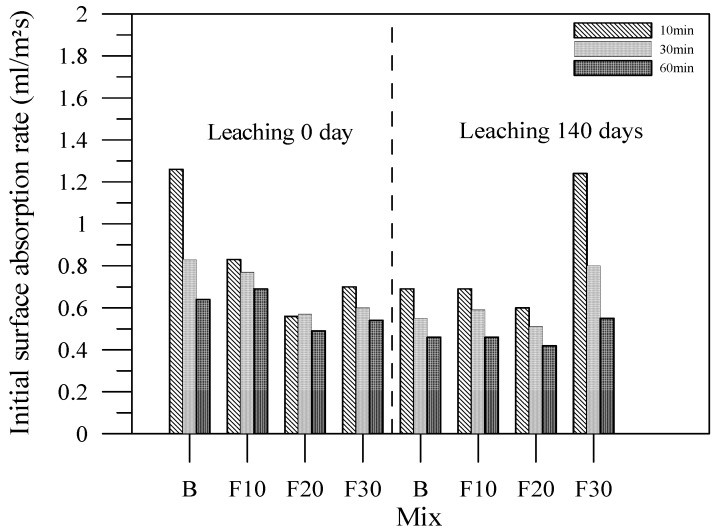
Initial surface absorptions of the specimens with fly ash admixtures.

The initial surface absorptions of the specimens with fly ash admixtures prior to leaching provide evidence that the microstructure of the cement-based materials can be significantly denser by replacing the fly ash admixture than the B specimens as shown in Figure 17.

**Figure 18:**
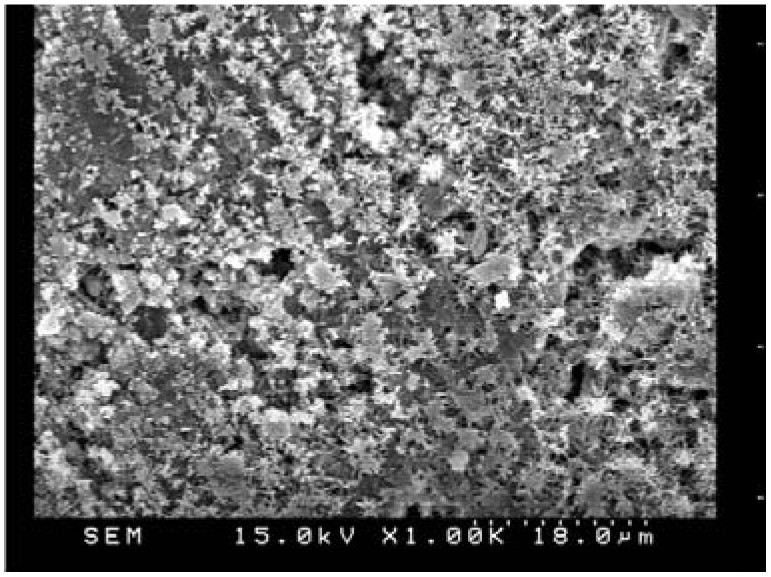
SEM micrograph of the cement-based material with fly ash admixture prior to leaching (×1 k).

**Figure 19:**
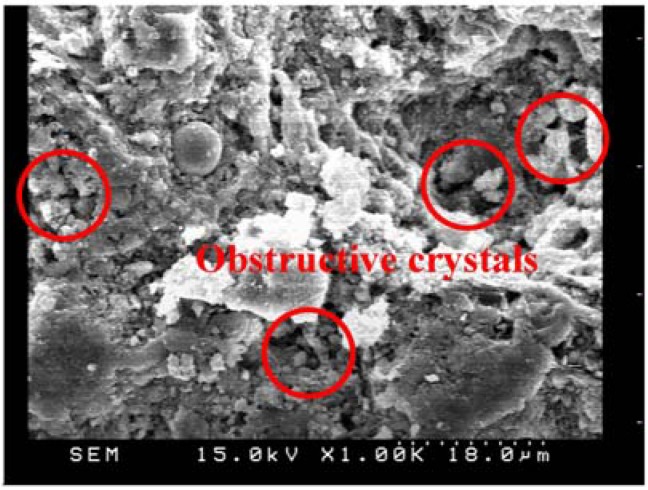
SEM micrograph of the cement-based material with fly ash admixture after leaching (×1 k).

After the leaching test for 140 days, the absorption rate of the specimens was lower than those before the leaching test except for F30 specimens. Such results may be explained by the calcium leached filling the pores of the surface zone. These hydration crystals filled the capillary pores of the surface layer on the specimens. Such mechanism may be responsible for the erroneous results of the initial surface absorption test, which is consistent with the previous study [17]. However, the results of the initial surface absorptions after leaching revealed that the leaching process resulted crystals of obstruction through observation of SEM, as shown in Figure 18 and Figure 19. Similarly, the specimens of cement-based materials with slag or silica fume share the same phenomenon with fly ash.

### 3.7. Mercury Porosity Test

**Figure 20:**
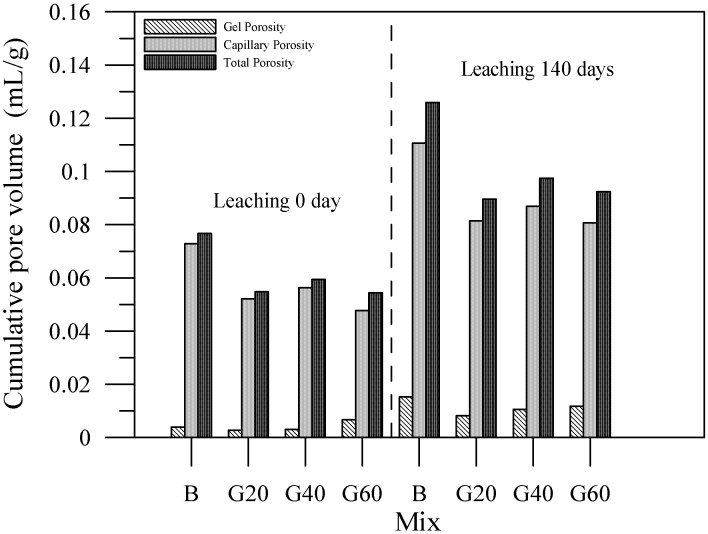
Porosity distribution of the specimens with slag admixture.

The mercury intrusion process can effectuate observations on the variations of gel porosity, capillary porosity, and total porosity of the specimen with an alkali activator. Figure 20 shows that the specimens with slag admixture can facilitate porosity reduction. Due to slag admixtures being able to produce C–S–H gels and slow the leaching of calcium ions, they withhold the tendency of porosity increase. The specimens of cement-based materials with fly ash or silica fume demonstrate the same phenomenon with slag.

**Figure 21:**
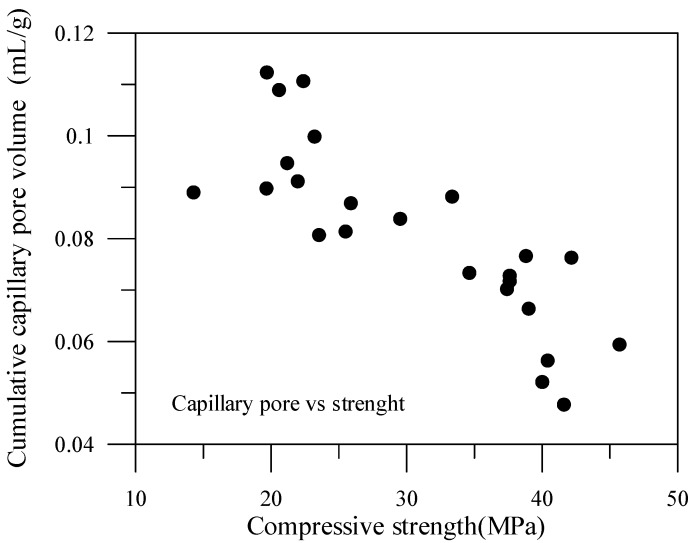
Compressive strength *vs.* capillary pore of specimens with mineral admixtures.

**Figure 22:**
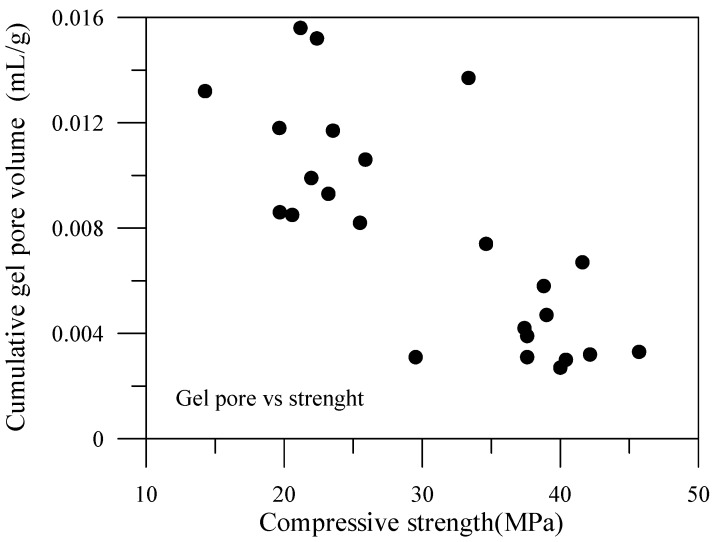
Compressive strength *vs.* gel pore of specimens with mineral admixtures.

The relationship of the compressive strengths and the pore volume of the specimen can consequently be plotted. The data for capillary pore volume and compressive strength are shown in Figure 21, while the data for gel pore volume and compressive strength are shown in Figure 22. The content of the capillary pores of the specimen are thus comparably high, and therefore, the capillary pore volume and compressive strength are relevant. In contrast, the content of gel pore volume is in a fairly low percentage, consequently having less correlation with compressive strength.

### 3.8. Ultrasonic Pulse Velocity Characteristics

**Figure 23:**
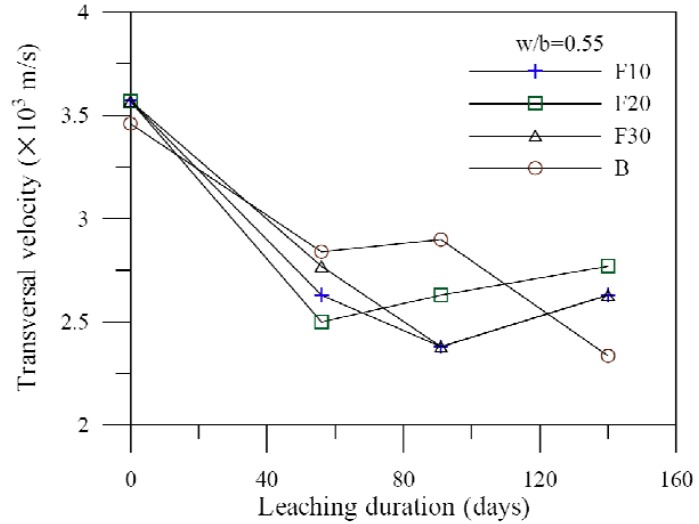
Ultrasonic measurement of the specimen with fly ash admixture *vs.* leaching duration.

The characteristics of wave propagation behaviors are in accordance with the transmission of waves with the transfer rate of different media, of which the denser medium provides increased velocity. Figure 23 illustrates the ultrasonic measurement of the specimen with the fly ash admixture at different leaching periods. The velocity decreases with an increase in leaching time. Furthermore, the specimens with the fly ash admixture can produce denser C–S–H gels to slow the leaching of calcium ions. Therefore, the velocity decreases more gently with an increase in leaching time. Similarly, the specimens of cement-based materials with slag or silica fume share the same results of those with fly ash.

## 4. Conclusions

This study illustrated the effects of leaching behavior of calcium ions on compression and durability of cement-based materials with various mineral admixtures. The conclusions are as follows:

1. Cement-based materials with mineral admixtures can facilitate trimming down the degradation process because an appropriate mineral admixture can produce C–S–H gels by consuming calcium hydroxide content. Due to denser C–S–H gels, the leaching process of calcium ions decelerates, slowing the deterioration of compressive strength of the specimens. Therefore, adding appropriate mineral admixtures into cement-based materials not only helps increase compressive strength, but also provides resistance to the leaching process from porosity generation. In brief, the mineral admixtures reduce calcium hydroxide quantity and refine pore structure via pozzolanic reaction, thus enhancing the compressive strength and durability of the cement-based materials.
2. The mineral admixture replacing cement results in a post-production pozzolanic reaction, resulting in the production of C–S–H gel, which is able to enhance specimen density, reducing the porosity in the specimen and also affecting the pore size distribution. The leaching depths of the cement-based material specimens with mineral admixtures over 140 days are less than those of specimens without mineral admixtures. Adding an appropriate amount of mineral admixture facilitates a reduction in the leaching depth of the cement-based material.
3. The SEM images showed that the hydration products of the cement-based material with mineral admixtures significantly decreased and resulted in increasing porosity. The XRD results of the specimens after leaching revealed that the specimens in the deterioration portion do not contain a calcium hydroxide component. The results of the Thermogravimetry analysis showed that the specimens with mineral admixtures can facilitate incrementation of the development of C–S–H gels while decreasing calcium hydroxide content.
4. The initial surface absorption test revealed that the specimens with mineral admixtures can facilitate porosity reduction. The experimental results show that the capillary porosity and gel porosity of the cement-based materials with mineral admixtures increased with the leaching process, based on mercury intrusion porosimetry, while the velocity decreased, based on ultrasonic pulse velocity.
